# Starvation selection reduces and delays larval ecdysone production and signaling

**DOI:** 10.1242/jeb.246144

**Published:** 2023-09-29

**Authors:** Jennifer M. Clark, Allen G. Gibbs

**Affiliations:** School of Life Sciences, University of Nevada, Las Vegas, 4505 S. Maryland Pkwy, Las Vegas, NV 89154-4004, USA

**Keywords:** Starvation, Ecdysone, Larval development, Body size, Evolution, Fat

## Abstract

Previous studies have shown that selection for starvation resistance in *Drosophila melanogaster* results in delayed eclosion and increased adult fat stores. It is assumed that these traits are caused by the starvation selection pressure, but its mechanism is unknown. We found that our starvation-selected (SS) population stores more fat during larval development and has extended larval development and pupal development time. Developmental checkpoints in the third instar associated with ecdysteroid hormone pulses are increasingly delayed. The delay in the late larval period seen in the SS population is indicative of reduced and delayed ecdysone signaling. An enzyme immunoassay for ecdysteroids (with greatest affinity to the metabolically active 20-hydroxyecdysone and the α-ecdysone precursor) confirmed that the SS population had reduced and delayed hormone production compared with that of fed control (FC) flies. Feeding third instar larvae on food supplemented with α-ecdysone partially rescued the developmental delay and reduced subsequent adult starvation resistance. This work suggests that starvation selection causes reduced and delayed production of ecdysteroids in the larval stage and affects the developmental delay phenotype that contributes to subsequent adult fat storage and starvation resistance.

## Teaser

**Summary:** Evolution from starvation selection can reduce and delay hormones that coordinate developmental transitions and extend the larval and pupal period in the fruit fly.

## INTRODUCTION

In the wild, periods without food are a common stressor that many animals encounter and must be able to survive in order to maximize their fitness. Starvation resistance of the adult fruit fly *Drosophila melanogaster* is thought to be primarily conferred by adult fat stores, body size, metabolic rate, and behavior or activity levels (Gibbs and Reynolds, 2012). Flies selected for adult starvation resistance show increased larval lipid accumulation and delayed pupariation (Chippindale et al., 1996). Altered larval development is usually indicative of altered hormone signaling as developmental timing is orchestrated by the steroid hormone ecdysone and many developmental genes are ecdysone responsive (Thummel, 2001). Development time is known to contribute to adult starvation resistance by affecting body size and nutrient stores (Chippindale et al., 1996; Kolss et al., 2009; Yan et al., 2015). Nutrition also regulates the timing of development by acting on growth of the steroidogenic organ (Layalle et al., 2008). Multiple nutrition and developmental cues converge on insulin and steroid signaling (reviewed in Texada, et al., 2020). Therefore, ecdysone signaling is a candidate target of starvation selection.

Canonically, there are three small peaks of ecdysteroid in the third instar that are coordinated with various developmental checkpoints. These were purportedly measured by Richards (1981) and Warren et al. (2006); however, the methodology for these assays lacked the precision, sensitivity and sample number to detect statistically definable ‘peaks’. New protocols were recently published which will hopefully aid in the elucidation of hormone titer levels during larval development (Koyama and Mirth, 2021), but ecdysone profiles using these new methods have not been published yet. Despite this, owing to information from gene expression analysis and other insects such as *Manduca*, it is widely accepted that small peaks of ecdysone are correlated with third instar developmental checkpoints.

The first developmental checkpoint occurs early after the transition to the third instar (Rewitz et al., 2013) and is correlated with the attainment of critical weight. Next, imaginal disc growth controls a transition that occurs near a change of gene expression, which can be visualized with a fluorescent gene marker. Lastly, larval wandering behavior is an indicator of the attainment of the final checkpoint of larval development.

Critical weight is assessed by the prothoracic gland (PG). Genetic and environmental differences such as nutrient status, nutrient signaling, temperature, photoperiod and PG size can change the timing and/or mass that determines critical weight (Mirth et al., 2005). After nutrition signals the attainment of critical weight, molecular machinery must commit the animal to hormone production and further development. Normal levels of hormone production that lead to on-time development depend on endocycling and chromosomal copy number in cells of the PG, but animals without endocycling can still develop and pupariate after about a 12 h delay (Ohhara et al., 2017; Shimell and O'Connor, 2023). When the PG is growth restricted by the inactivation of insulin signaling in whole larvae, it can extend the larval period by delaying prothoracicotropic hormone (PTTH) release to allow time for the larvae to reach the same final body size (Shingleton et al., 2005). When PG growth is restricted by the inactivation of insulin signaling in the PG only, the PG delays PTTH release and extends larval development, but the rest of the organism can still respond to the growth-promoting effects of insulin, resulting in larger final body size (Mirth et al., 2005).

The next developmental transition associated with ecdysone occurs in the mid-third instar about 20 h after L3 (Warren et al., 2006) and is associated with several specific ‘chromosomal puffs’, which are regions of the chromosome that are being highly transcribed. These regions are ecdysone responsive, indicating large-scale genetic reprogramming by the hormone (Ashburner, 1972). For example, the mid-third instar transition marks the change in sensitivity to developmental delay in response to injured or slow growing discs secreting *Drosophila* insulin-like peptide 8 (Dilp8) (Hackney et al., 2012). Dilp8 can reduce ecdysone by downregulating the production of PTTH through the receptor Lgr3 on growth coordinating Lgr3 (GCL) neurons in the brain (Colombani et al., 2015; Garelli et al., 2015). Growing discs also secrete Decapentaplegic (Dpp) and can downregulate ecdysone production via the receptor Thickveins (Tkv) and by regulating insulin production via FOXO and *bantam* (Setiawan et al., 2018). Therefore, altered regulation, assessment or signaling of disc growth could potentially change larval nutrient stores, final body size, developmental time and subsequent adult starvation resistance. Evidence of these types of changes may be found in the timing of the mid-third instar transition.

The next developmental transition, wandering behavior, is associated with an increase in the rise of ecdysone and is normally gated by the photoperiod (Varma et al., 2019). Nutrition sensing also controls wandering behavior through regulation of ecdysone production via *pickpocket*-expressing neurons that use the amino acid sensor *slimfast* to detect arginine as a proxy for diet quality (Jayakumar et al., 2016; Wegman et al., 2010).

The starvation-selected (SS) population is an outbred population of starvation-selected *D. melanogaster* that has extreme starvation resistance, greatly increased triglyceride (TG) stores, and takes about one day longer to develop from egg to eclosion. The phenotype of the SS population indicates they are likely to have altered ecdysone signaling. Larval growth and attainment of developmental checkpoints were assayed and compared with those of an unselected fed control (FC) population that was derived from the same outbred starter population and maintained in parallel. For developmental assays, an inbred lab stock (LS) population (Canton-S) was included as an additional control to demonstrate the validity of the FC population as being representative of *D. melanogaster*. Levels of ecdysone during the third instar were investigated and the outcomes of ecdysone supplementation on the SS and FC populations were measured.

**List of abbreviations**

20E: 20-hydroxyecdysone
ALH: after larval hatching
CW: critical weight
FC: fed control
GFP: green fluorescent protein
LS: lab stock (Canton-S population) of *Drosophila*
PG: prothoracic gland
PTTH: prothoracicotropic hormone
Sgs3: salivary gland secretion 3
SS: starvation-selected
TG: triglyceride

## MATERIALS AND METHODS

### Starvation-selection regime

We studied populations of *Drosophila melanogaster* Meigen 1830 that have been selected for starvation resistance since 2007. The experimental evolution regime involves starving outbred, genetically heterogeneous adult flies on agar-only medium that provides water but no nutrition until only approximately 20% are left alive or up to 14 days. The surviving flies are reintroduced to food and allowed to recover before their eggs are collected to seed the next generation that will undergo the same process. The control and experimental populations in this experiment originate from wild-caught, outbred female flies collected in Terhune, New Jersey in 1998. SS and FC populations have been kept in triplicate as independent biological replicates (A, B and C). All lines have been handled in parallel and kept in large numbers of about 10,000 individuals per population. The three separate FC populations always have access to food. At the start of the experiment, the mean starvation survival time for all six populations was about 3 days. After 130 generations of selection, SS populations survived for an average of 10 days without food, with some individuals surviving for 19 days. FC populations still display a survival time of 3 days without food. The SS_A_ replicate has a higher mean starvation resistance for females (10.5 days) than that of the other replicates SS_B_ (8.2 days) or SS_C_ (8.5 days) (Hardy, 2016). Because of the labor-intensive nature of the developmental assays we performed, the experiments here used only the SS_A_ and FC_A_ populations.

### Animal housing

Adult flies from the inbred laboratory stock Canton-S and the SS and FC populations one generation removed from selection (F1) were housed in large Plexiglas cages with several thousand individuals. These animals were maintained in an incubator at 25°C with 24 h light and ambient humidity (approximately 25–45%). Food was provided by two Petri dishes containing sucrose–yeast–cornmeal medium, which were replaced every other day. A dish with a cotton ball soaked with water was also placed inside the cage to provide additional humidity and an extra water source. The 24 h light regime was chosen so as to facilitate continual egg laying as opposed to the crepuscular behavior pattern. Therefore, there were no light cues to entrain the animals.

### Egg collection and staging larvae at hatching

Molasses agar (10% molasses and 3.4% agar) with yeast paste (approximately 1:1 dry active yeast mixed with water) was given to the cages to condition F1 adults for egg laying with this nutrient-rich food for 24–48 h. This ‘priming’ period increases egg production and habituates the animals to the new egg laying substrate. To collect eggs, molasses agar plates with yeast paste were presented in 4 h intervals resulting in cohorts of F2 eggs. These dishes were kept at 25°C under 24 h constant light and ambient humidity (25–45%) as described above. About 21 h later, any early-hatching larvae were cleared from the molasses agar plates. One hour after clearing, newly hatched larvae were collected for 1 h at low density in groups of 25 (Baldal et al., 2005) and placed into vials of food, and then aged in an incubator at 25°C under 24 h constant light and ambient humidity. Therefore, at the time of collection, these animals were 1 h (±1 h) after larval hatching (ALH).

### Larval staging assay (L1–L2 and L2–L3 molt)

In 1 h intervals during expected molting periods between larval instars, vials of larvae collected in the manner above had their food plug pulled out onto a dish under a dissecting microscope and as many animals as could be collected in about 10 min were then scored by their developmental stage. This was repeated for each population. The morphology of the mouth hooks and the anterior spiracles was used to assess the larval stage. The transitions between larval instars were defined by the time at which 50% of the cohort had molted to the later instar.

### Wandering, pupariation and eclosion assays

Vials of larvae collected in the manner above (±1 h old) were left unperturbed on a 24 h:0 h light:dark cycle to remove synchronization by light cues. These vials were used to visually score the animals every 4–6 h as they progressed through the wandering stage, the white pre-pupae stage (WPP) and the transition to brown pupae. Adults were collected as they eclosed in 8–12 h intervals, briefly anesthetized on a CO_2_ pad and scored for sex.

### Probit analysis for larval molts, wandering, pupariation and eclosion assays

A probit analysis was used to evaluate the developmental data. The hours of development were compared with the all-or-nothing (quantal) response of whether the animal had transitioned into the next stage or not. The stimulus–response probability was calculated for each stimulus ‘dose’ (hours of development) assayed and the average probability of pooled data from three independent experiments was plotted. Third-order polynomials were used to determine the development time at which 50% of animals had undergone the transition to the next stage.

### Pupal duration (restaging at WPP)

To resynchronize the development of the animals at the WPP stage, every 2 h new WPP were delicately transferred to a fresh vial using a paintbrush. Their eclosion was scored in 8 h intervals beginning at 8 days ALH, resulting in a measure of pupal duration.

### Critical weight and viable weight behavioral transition timing

Beginning 2 h after L3 for the FC population (verified by staging this cohort in the manner described above), L3 larvae were collected in 2 h intervals from both populations from an undisturbed vial and washed in phosphate-buffered saline to clean off any food particles. The larvae from each population were split into either fed or starved treatment groups. The fed treatment group was transferred in groups of five larvae to at least each of three vials of regular fly food, whereas the starved treatment group was transferred similarly to vials of 1% non-nutritive agar. An additional group was frozen to have its TG and protein content measured. The minimum time animals must feed (in h after larval hatching) before being able to pupariate without delay upon starvation compared with unstarved controls was determined as the timing of critical weight. The minimum time animals must feed (in h after larval hatching) that results in at least 50% eventual pupariation was determined as minimum viable weight.

### Transgenic SgsΔ3-GFP population foundation

The SgsΔ3-GFP construct (Biyasheva et al., 2001) contains the regulatory regions and N-terminal region of the endogenous salivary gland secretion 3 (Sgs3) or ‘glue’ protein fused with the coding information for enhanced green fluorescent protein (GFP). It was on a pCasper4 plasmid (stored and provided by Andrew J. Andres; Biyasheva et al., 2001) transformed into *Escherichia coli*. The plasmid was verified by DNA gel electrophoresis and a culture was isolated and sent to RainbowFly Transgenic Flies for injection into F2 embryos of SS and FC flies. The injected embryos were returned and the resulting F2 adults were individually crossed to either males or females of their population of origin. The F3 progeny were screened under a fluorescence microscope for glowing salivary glands that were expressing SgsΔ3-GFP. The rate of insertion is usually 10–20% for assisted P-element transgenesis in typical laboratory strains (Engels, 2001). Successfully transformed F3 progeny were recovered from three different crosses (two from FC, one from SS) out of about 50 crosses per population. Each group was collected and allowed to self-cross as it is assumed that the parent had only one unique insertion of the construct. GFP-positive F4 progeny were selected to establish a GFP-enriched population that will perpetuate through self-crossing. The crosses were monitored every generation for five generations to ensure that the GFP-containing chromosome was not being negatively selected against. Only the most robust FC-GFP transgenic population was chosen to be maintained.

### SgsΔ3-GFP expression

Female virgins from the SS-GFP and FC-GFP (transgenic populations with the SgsΔ3GFP insertion) were collected and crossed to males from the SS and FC populations. This pairing was determined necessary because the GFP insertion in the SS-GFP population appeared to be inserted on the X chromosome given its observed sex-specific segregation. Larval cohorts were staged and reared in the manner detailed previously. In 4 h intervals, animals were collected from the food, rinsed and placed under the fluorescence imaging microscope to be screened for the presence of GFP.

Because heterozygous GFP animals cannot be separated from homozygous GFP animals, some progeny from the crosses will inherit a non-transformed chromosome from both parents and will not have a transgene to express GFP. Therefore, after counting the number of glowing larvae in a sample, it was necessary to screen them again 12 h later for the total number of larvae/pupae that had the transgene. The time at which 50% of larvae expressed GFP relative to the total (final) number that went on to express GFP was used to indicate the timing of 20-hydroxyecdysone (20E) signaling.

### Fly homogenates and TG and protein assays

Flies previously frozen at −80°C were put in pairs into 2.0 ml tubes with a metal bead and 250 µl cold lysis buffer (100 mmol l^−1^ NaCl, 2 mmol l^−1^ MgCl_2_, 0.1 mmol l^−1^ CaCl_2_, 1% NP-40, 0.5% deoxycholic acid, 0.1% Triton X-100, pH 7.6). Cold bead-beater frames with samples were loaded into a Qiagen TissueLyser for 1 min at 20 Hz. Samples were then heated for 5 min at 70°C in a water bath for protein inactivation. Then, samples were centrifuged for 3 min at 13,700 ***g*** and 150 µl of the supernatant was collected into new 1.7 ml tubes without disturbing the pellet. Then, only the SS samples were diluted twofold by adding the same volume of Milli-Q H_2_O as the volume collected (150 µl). The Infinity TG Reagent Kit from Thermo Fisher Scientific was used to measure total TGs. In 96-well plates, 100 µl of reagent was pipetted into each well along with 10 µl of sample or standard. Plates were incubated for 15 min at 37°C and then read at 540 nm.

For the bicinchoninic acid (BCA) protein assay (reviewed in He, 2011), whole-fly homogenates from the same preparation as the TG assay were further diluted using Milli-Q H_2_O to a final 8× dilution for both populations. In 96-well plates, 200 µl of BCA reagent (Thermo Fisher Scientific) was pipetted into each well along with 10 µl of sample or standard. Plates were incubated at room temperature overnight and then read at 562 nm.

### α-Ecdysone feeding

Third instar SS and FC larvae synchronized at hatching were aged on normal food at 25°C under constant light in the manner described above. At 52 h ALH, in triplicate per population and treatment, 25 larvae were collected from the food and transferred to either treatment vials or mock control vials. Both types of vials had 3 ml of food on top of 10 ml of non-nutritive agar (to prevent the small volume of food from drying out). The food in the treatment vials had 0.5 mmol l^−1^ α-ecdysone (VWR) in an ethanol vehicle added (Ono, 2014), whereas the mock control vials only had the ethanol vehicle added. Vials were assayed for the time to pupariation and then newly eclosed adults were collected and subjected to a starvation resistance assay or frozen for subsequent fat content or protein content assays.

### Ecdysteroid quantification by enzyme immunoassay

Larvae staged at hatching as previously described were collected in three replicates of 10 animals for the later stages (84–112 h ALH) where the animals were larger and had more hormone to measure, and three replicates of 20 animals for the early stages (52–80 h ALH) when the larvae were very small. Results were calculated in pg per larvae to allow comparison. Larvae were collected from each population at 4 h intervals during L3. Larvae were washed in distilled water twice, briefly dried on paper towels and placed into ice cold methanol and kept at −80°C until use (Koyama et al., 2014). Prior to assaying, the samples were homogenized and centrifuged, the supernatant collected and the methanol from the supernatant was evaporated until completely dry (Mirth et al., 2005). Samples were resuspended in 100 μl enzyme immunoassay (EIA) buffer from the kit. EIA assay was performed as per the instructions of the 20E EIA kit purchased from Cayman Chemicals.

## RESULTS

### Starvation selection results in developmental extension that is primarily in the third larval instar

Ecdysone deficits manifest as delays in development or developmental arrest (failure to pupariate or molt) primarily in the third instar (Berreur et al., 1979; McBrayer et al., 2007). Characterizing the distribution of the developmental delay of SS *Drosophila* could implicate ecdysone signaling deficiency as the cause of the developmental period extension and therefore validate ecdysone signaling as a potential target of starvation selection. We measured the development time of each larval stage, eclosion and larval transitions beginning with all animals staged at hatching.

#### Eclosion

**Fig. 1.**
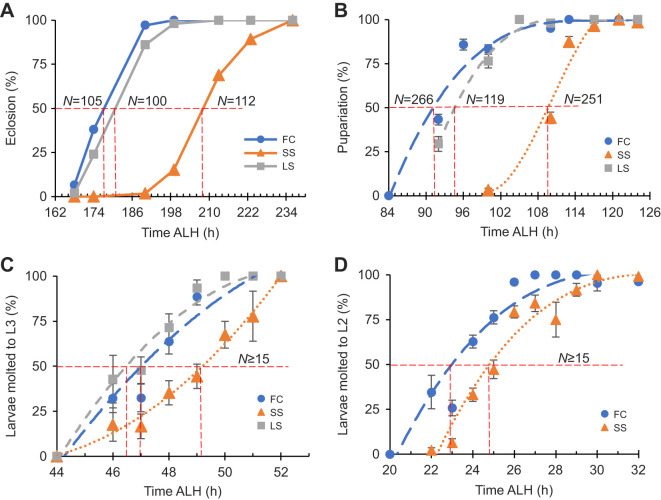
**Developmental stage transitions occur later in a starvation-selected population.** (A) Line chart showing eclosion rates of pooled females and males against development time for three populations, fed control (FC), starvation-selected (SS) and the ‘lab stock’ (LS) Canton-S. A red dashed line is plotted along the 50% line and drawn down to the *x*-axis at the intersection with the plotted data from one representative experiment. (B–D) Data points represent predicted probabilities of (B) pupariation, (C) L3 molt or (D) L2 molt, based on hours of development using a model generated from a probit analysis of data from three independent experiments. Error bars are standard error of the predicted probability. Trendlines are third-order polynomials. (C,D) *N*≥15 larvae per time point per population per experiment. LS was not measured in D. ALH, after larval hatching.

The SS animals (females and males pooled) eclosed (mean±s.e.m.) 33.7±0.13 h (±1 h) after the FC animals, which eclosed 7.5±0.07 h (±1 h) before the LS animals (Fig. 1A), indicating that the SS population is much different from the FC and LS populations, which are similar to each other.

#### Pupal period

Although pupal period can be inferred indirectly from the timing of pupariation and the timing of eclosion, it was measured directly by restaging animals at WPP and collecting them upon eclosion. The pupal period was shown to be extended in the SS population by 10±4 h in Fig. S1.

#### Pupariation

Pupariation occurred 15.6±0.11 h (±1 h) later in the SS population than in the FC population (Fig. 1B). The LS population was similar to the FC population, pupariating just 3.5±0.09 h (±1 h) later.

#### Larval molts

As it appears that the majority of the extra developmental time is spent in the larval period, we asked during which larval stage this occurs. Time to L2 and L3 was assayed. Fifty percent of SS larvae had molted to L3 2.25±0.08 h (±1.25 h) later than the FC larvae (Fig. 1C). The LS and the FC larvae showed no difference. The window of ±1.25 h comes from the age of the animals (±1 h) and the duration of the assay (0.5 h, or ±0.25 h). Time to 50% probability of second instar molt was 1.75±0.15 h (±1.25 h) delayed in the SS population (Fig. 1D). Time to hatching was not measured as preliminary experiments indicated that the delay was smaller than the staging window (data not shown).

### Starvation selection delays third instar larval checkpoints associated with ecdysone signaling

#### Critical weight and viable weight

Critical weight is the mass at which a larva is committed to pupariation in the absence of additional food (reviewed in Mirth and Shingleton, 2012). Larvae that have attained critical weight will accelerate their time to pupariation to escape larval starvation. Viable weight occurs just before critical weight in *Drosophila*. Viable weight occurs when the larva is competent to pupariate but, upon starvation, will delay pupariation in search of more food, before eventually pupariating later than animals that were not starved.

Measuring the competence of the SS larvae to pupariate in the absence of additional food will indicate the timing of their attainment of this checkpoint. It should be noted, however, that the actual mass of the larvae that this competence coincides with was not specifically measured. Growth curves for larval lipid and protein accumulation are provided for additional context for these results but these are whole-body TG and protein measurements and not from the same individuals that underwent measurement of critical weight.

**Fig. 2.**
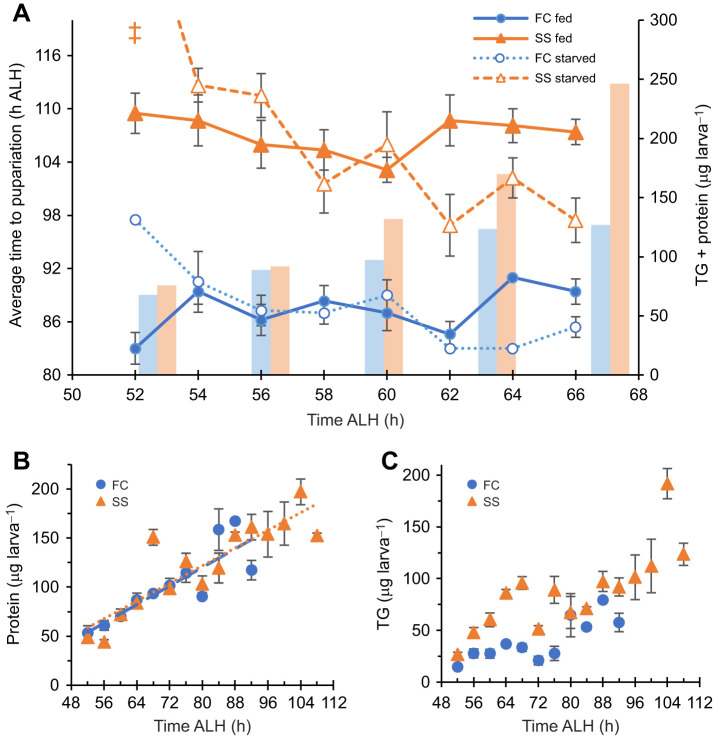
**Critical weight is not delayed in the SS and FC population.** (A) Average time to pupariation for either FC or SS after being transferred back to food (handling control treatment, filled symbols) or non-nutritive agar (open symbols) at different times in development along the *x*-axis. Each point represents the mean pupariation time of at least five animals and error bars represent standard error of the mean. Trend lines are the moving average. The symbol ‡ indicates that these animals never pupariated. Superimposed bar graph represents protein plus triglyceride (TG) levels, which are independently graphed in B and C. Three biological replicate samples were each measured three times for protein and TG at each time point and for each population in B and C.

The timing of critical weight (but not the exact mass) was measured by starving third instar larvae at intervals during early third instar. The FC and SS populations both consistently pupariated on time or ahead of refed controls when starved at 62±2 h ALH or after, indicating that critical weight was achieved (Fig. 2A). The SS population was not delayed in attaining this competency to pupariate under starvation, indicating that the observed developmental delay was not due to altered critical weight timing. The SS population animals do not appear to have an undergrowth phenotype, as their protein and TG levels were not reduced compared with those of the FC population (Fig. 2B,C).

#### SgsΔ3-GFP expression

**Fig. 3.**
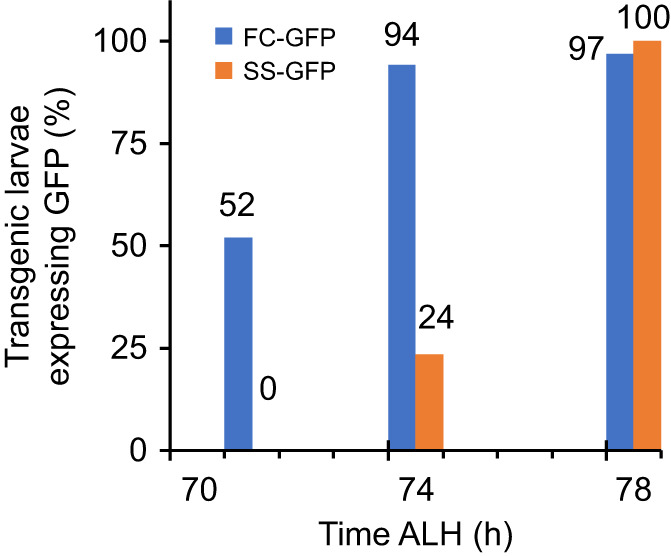
**SgsΔ3-GFP expression is delayed in the SS-GFP population.** (A) Bars show the percentage of larvae expressing SgsΔ3-GFP in their salivary glands out of the total number of larvae that went on to express SgsΔ3-GFP, as identified by fluorescence microscopy. One representative experiment is shown. *N*=10, 8 and 10 animals for FC-GFP, and *N*=11, 15 and 14 animals for SS-GFP.

We made a transgenic population of the SS and FC populations to include a reporter for mid-third instar ecdysone signaling. The FC-GFP and SS-GFP transgenic populations created have fluorescent reporters attached to salivary gland glue proteins that are expressed in response to the mid-third instar pulse of ecdysone. This existing tool (Biyasheva et al., 2001) was transformed into the SS and FC populations to visualize the expression of salivary gland glue genes that indicate the timing of the developmental transition coordinated by ecdysone signaling in the mid-third instar. A fluorescence image of an *in vivo* larva expressing the GFP transgene in the salivary glands can be seen in Fig. S2E. The SS-GFP population reached 50% expression 5±3 h after the FC-GFP population (Fig. 3). These derived populations were first confirmed to recapitulate the relevant phenotypes of the parental population (Fig. S2A–D).

#### Wandering

**Fig. 4.**
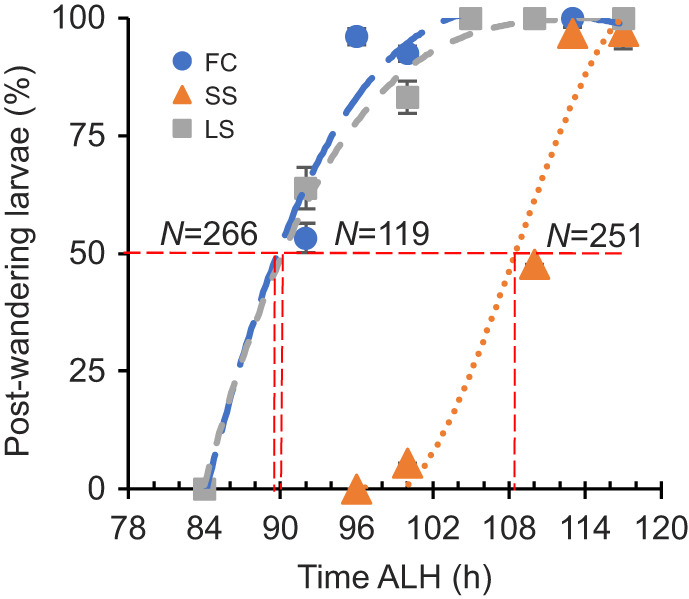
**Larval wandering occurs later in the SS population.** Data points represent predicted probabilities of wandering based on hours of development using a model generated from a probit analysis of data from three independent experiments, each with *n*>100 animals per population assayed at each time point. The SS population wander 19±4 h later. Error bars are standard error of the predicted probability. Trendlines are third-order polynomials.

Wandering is a behavior that is defined by the larvae crawling out of the food and up the walls of the vial in preparation for pupariation. It took the SS population 19±4 h longer to reach 50% wandering compared with the FC population, whereas the LS population was similar to the FC population, taking only 2±4 h longer (Fig. 4).

#### Development time summary

**Fig. 5.**
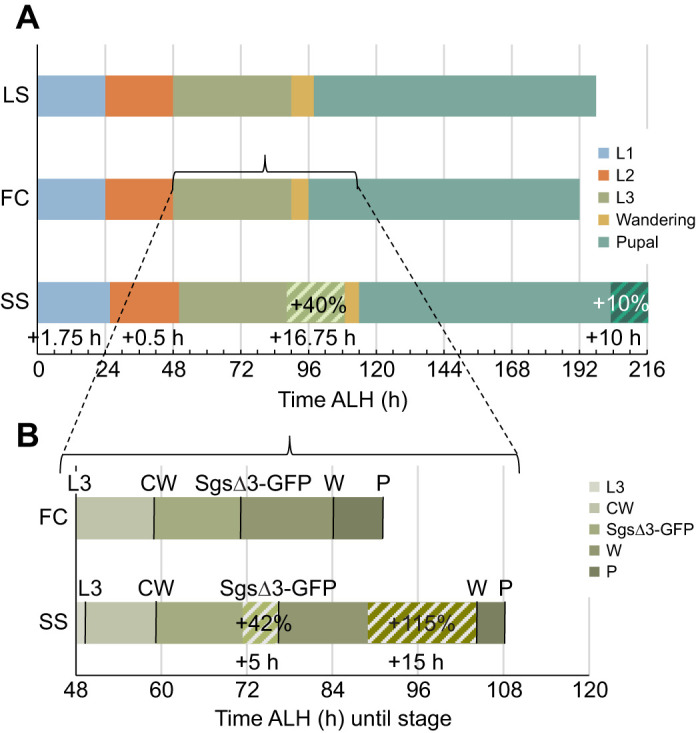
**Partitioning of developmental delay in the SS population.** (A) Horizontal stacked bar graph shows the duration of each stage for the SS and LS populations compared with that for the FC population. Only the stages with the greatest difference in the SS population are visually differentiated to show magnitude. (B) Stacked bar graph of FC and SS populations hours after larval hatching until each third instar physiological stage of development. Delayed attainment of a stage is represented by a larger bar. The *x*-axis begins at L3 transition of the FC population; therefore, the corresponding colored bar does not appear. CW, critical weight; P, pupariation; W, wandering.

 Fig. 5A illustrates the time spent in the various stages for the FC, LS and SS populations. Achieving pupariation (time spent in L3) took 40% longer in the SS population and pupal duration was increased by 10%. As the largest delay is in the third instar, Fig. 5B shows the third instar on an expanded scale and bars show the time until the labeled developmental transition was reached (as the developmental transitions are not stages per se). Achievement of the wandering stage (i.e. late third instar) is the most greatly delayed transition assayed in the SS population.

### Starvation selection is associated with delayed ecdysone peaks

#### 20E enzyme immunoassay

To discern between reduced hormone availability and blunted hormone response, the ecdysteroid titer was measured at short intervals during the larval third instar using a 20E enzyme immunoassay (EIA) that measures 20E and its related metabolites, including α-ecdysone at varying affinities. The greatest sensitivity is primarily towards the most metabolically active metabolites (Table S1).

**Fig. 6.**
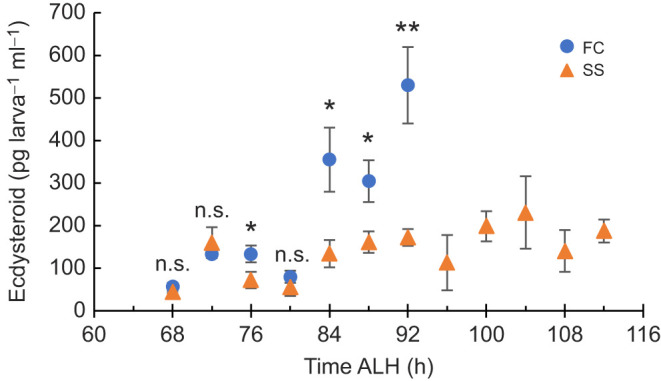
**Ecdysteroid levels remain low longer in the SS population.** Data points represent the average of three biological replicate values for the 20E enzyme immunoassay (EIA). Three groups of *N*=20 for time points 68–80 h ALH and *N*=10 for 84–112 h ALH were measured at each time point for each population. Results are reported in pg per larva. Error bars represent standard error. Significantly different values as returned by a *t*-test are indicated by asterisks (*P<0.05 or **P<0.01); n.s., not significantly different.

The hormone pulses rose later in the SS population compared with the FC population (Fig. 6; Fig. S3). Hormone titers remained low in the SS population after the FC population had already mounted the pupariation peak. This indicates that hormone production is reduced and delayed under starvation selection.

### Larval ecdysone exposure and life history contribute to starvation resistance

#### Feeding exogenous hormones partially rescues the SS development time phenotype

**Fig. 7.**
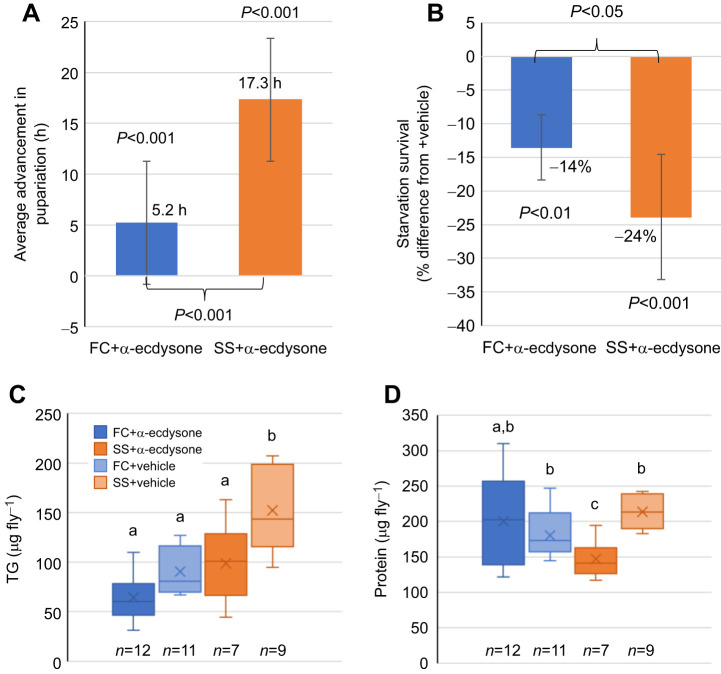
**Exogenous hormone feeding partially rescues SS phenotype.** (A) Average advancement of time of pupariation for animals fed on α-ecdysone supplemented food at 52 h ALH versus food with vehicle alone from three independent experiments. *N*=75 for each group. (B) Percentage difference of adult starvation survival duration (h) following hormone treatment versus vehicle treatment for each population. (C,D) Boxplots showing (C) triglyceride or (D) protein stores of newly eclosed adults for FC or SS larvae that were reared in the same experiment. *n* represents number of pairs of flies assayed, letters represent significance groups as returned by a Tukey *post hoc* test following a one-way ANOVA for each panel, using *P*<0.05.

Third instar larvae were reared on food containing exogenous hormones to attempt to rescue the delayed development phenotype and further test the hypothesis that reduced or delayed ecdysone signaling is responsible for the delay. α-Ecdysone was able to accelerate pupariation in both the FC [mean±95% confidence interval (CI), 5±1.8 h (±6 h)] and the SS [18±1.8 h (±6 h)] populations (Fig. 7A). 20E was also preliminarily tested (Fig. S4). Although it accelerated time to pupariation, it did not have a statistically significant effect on subsequent adult starvation resistance at the concentrations used (see Supplementary Materials and Methods). Owing to the cost of the 20E reagent, further experiments were conducted with α-ecdysone at different concentrations.

#### Feeding exogenous hormones reduces adult starvation resistance

To measure the effect of larval hormone exposure on adult starvation resistance, the same animals exposed to α-ecdysone or vehicle-only treatment and measured for development time were also subjected to starvation upon eclosion (Fig. 7B). Average starvation resistance was reduced in the SS population by a greater amount and a greater percentage relative to that in the vehicle-treated population (mean±95% CI, 24±9%) than that in the FC population (14±5%). The difference between the averages was significant (*P*<0.05), indicating that the SS population had a different response to the exogenous hormone.

#### Feeding exogenous hormones alters adult body composition

After larval exposure to α-ecdysone or vehicle alone, adult body composition was measured upon eclosion. Whole-body TG and protein were measured (Fig. 7C,D). TG was reduced in the SS+α-ecdysone treatment compared with that in the vehicle-alone treatment (*P*<0.05) and was statistically similar to that in the FC both with and without hormone treatment (*P*>0.05). Protein level in the SS population was also reduced by the hormone treatment versus the vehicle treatment and it was also significantly less than that in either treatment of the FC population (*P*<0.05).

## DISCUSSION

The distribution of extra developmental time in the SS population was determined by comparing developmental transitions with those of the FC population (which itself was confirmed to be similar to the LS; Canton-S) in order to generate evidence for hypotheses regarding the cause of delayed development. Most of the extra developmental time occurs in the third larval instar, which is consistent specifically with delayed or reduced ecdysone signaling. The extended development time is inconsistent with alternative explanations, such as growth rate reduction, based on the measure of larval body protein and TGs. The timing of critical weight attainment was not delayed (though the mass at critical weight was not measured); however, attainment of developmental milestones dependent on ecdysone production was increasingly delayed. Whole-body ecdysteroid levels were measured and found to be reduced and delayed in the SS population. Exogenous application of the hormone α-ecdysone beginning in the early third instar was able to partially rescue the delayed development of the SS population, confirming that reduced ecdysone signaling is a contributing mechanism for the phenotype. Further, exposure to this treatment impacted subsequent adult TG and protein levels and starvation resistance, and the SS population was more sensitive to this effect.

Critical weight was attained at the same time in the SS population as in the FC population (Fig. 2A); however, the SS animals already had greater fat stores at this time (Fig. 2C). Fat accumulation was accelerated in the SS population during the larval stage, consistent with Brown et al. (2019), showing that, during the third instar, the SS population consumed more food and decreased its metabolic rate compared with the FC population. However, protein accumulation appears to be at the same rate (Fig. 2B), so this could be an interesting model to elucidate the inputs to critical weight in *Drosophila*.

Mid-third instar development was assessed with the genetic tool SgsΔ3-GFP (Fig. 3) but not directly, such as through imaginal disc size and staging. How starvation selection affects disc growth and whether any changes are due to factors other than altered ecdysone signaling would be an area of interest.

The largest portion of extended development was spent in the late third instar before achieving wandering; however, the duration of wandering itself was actually an average of about 3 h shorter in the SS population compared with that in the FC population (summarized in Fig. 5B). The significance of this consistent finding was not addressed in this work but may be informative for future hypothesis generation.

The limitations of these experiments were that no statistically definable ‘peaks’ of ecdysteroids were observed because the animals used for ecdysteroid measurements were not restaged at the third instar as in the protocol from Koyama and Mirth (2021). Experimentally, we chose not to restage animals because of the delayed development occurring before the third instar in the SS population. Additionally, the ecdysteroid levels cannot be directly mapped to the timing of developmental checkpoints tested because they were not measured on the same cohorts of animals.

Identifying reduced and delayed ecdysone production as a consequence of starvation selection confirms many previous observations, but it does not address why reduced ecdysone signaling may be supportive of starvation resistance. An intuitive hypothesis would be that a longer development time affords the animals more time to grow and, therefore, more stored nutrients in reserve for adult starvation selection. However, there is also evidence that extended larval development itself is not supportive of starvation resistance or stress tolerance, but merely a secondary effect of perturbed insulin and insulin-like signaling (Zwaan et al., 1991; Broughton et al., 2005). Pointedly, it has repeatedly been observed that reduced metabolic rate is a key factor for starvation resistance in the SS population (Brown et al., 2019) and that SS flies of the same weight as FC flies still survive starvation better (Hardy, 2016). Therefore, cause and effect are not distinguished by these experiments alone.

It must also be considered that ecdysone controls many functions in the adult in addition to its well-known role in coordinating larval development. Adult ecdysone signaling modulates traits that could potentially support starvation resistance, including fecundity (Meiselman et al., 2017), activity (Kumar et al., 2014), behavior (Brewer, 2013; Schwedes et al., 2011) and sleep (Brown et al., 2019; Ishimoto and Kitamoto, 2010; Masek et al., 2014; Slocumb et al., 2015). It has always been intriguing how the adult selection pressure on the SS population has produced a larval phenotype; however, it is plausible that ecdysone signaling in the adult was the target of selection and a global reduction also affected the larval stage. Additionally, ecdysone production itself might not be a direct target of starvation selection but may likely result from another cause such as altered nutrition sensing or signaling by the fat body or PG that inhibits ecdysone production as a downstream effect, such as effects on PTTH production (Shimell et al., 2018).

Previously known mechanisms of coordinating development that are associated with the pattern of delays observed in this work warrant further investigation, particularly PG size and body size assessment, Dilp8 signaling, imaginal disc growth, and amino acid-sensing neurons controlling development. Additionally, detecting potential changes in the mass at critical weight may be of interest, as well as the response to feeding a mixture of 20E and α-ecdysone. Further, exposure to exogenous hormones through the water source during adult starvation in the SS population may allow us to explore the role of ecdysone during adult starvation. Investigating these and other mechanisms by which starvation selection imparts starvation resistance and its associated phenotypes, such as increased TG stores and extended larval development, might reveal interactions in physiological systems that can be used to inform research on human puberty and obesity, given the orthologous nature of fruit fly and mammal fat metabolism (Trinh and Boulianne, 2013), nutrition signaling (Rajan and Perrimon, 2013) and developmental hormone signaling (Hyun, 2018; Niwa and Niwa, 2011; Yamanaka et al., 2015; Klinge, 2018) at the cellular level.

## Supplementary Material

10.1242/jexbio.246144_sup1 Supplementary information
