# Supplementary material for: Starvation selection reduces and delays larval ecdysone production and signaling
Source: J Exp Biol. 2023 Sep 29;226(18):jeb246144. doi: 10.1242/jeb.246144 (PMC10560552; doi:10.1242/jeb.246144)
Supplement: Supplementary information [file jexbio-226-246144-s1.pdf]

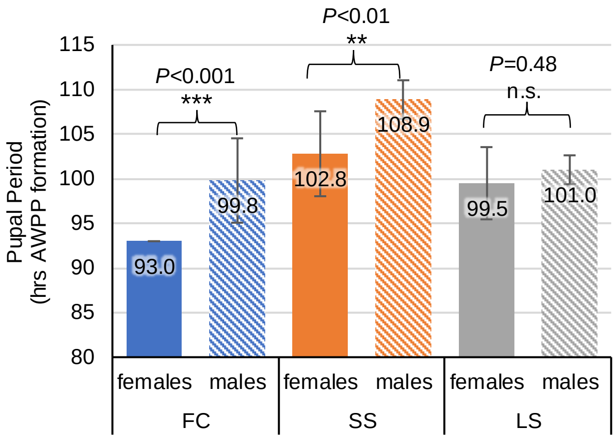

Fig. S1. Pupal period duration differs by sex and selection.

Bar graph of average pupal period duration of animals staged at WPP, separated by sex and population. N for females of the FC, SS, and LS population are N=14, 5, and 8 and males N=10, 8, and 6. Multiple t-tests were performed between populations to return Bonferroni-corrected significance values. Error bars are 95% confidence interval of the mean.

Journal of Experimental Biology • Supplementary information

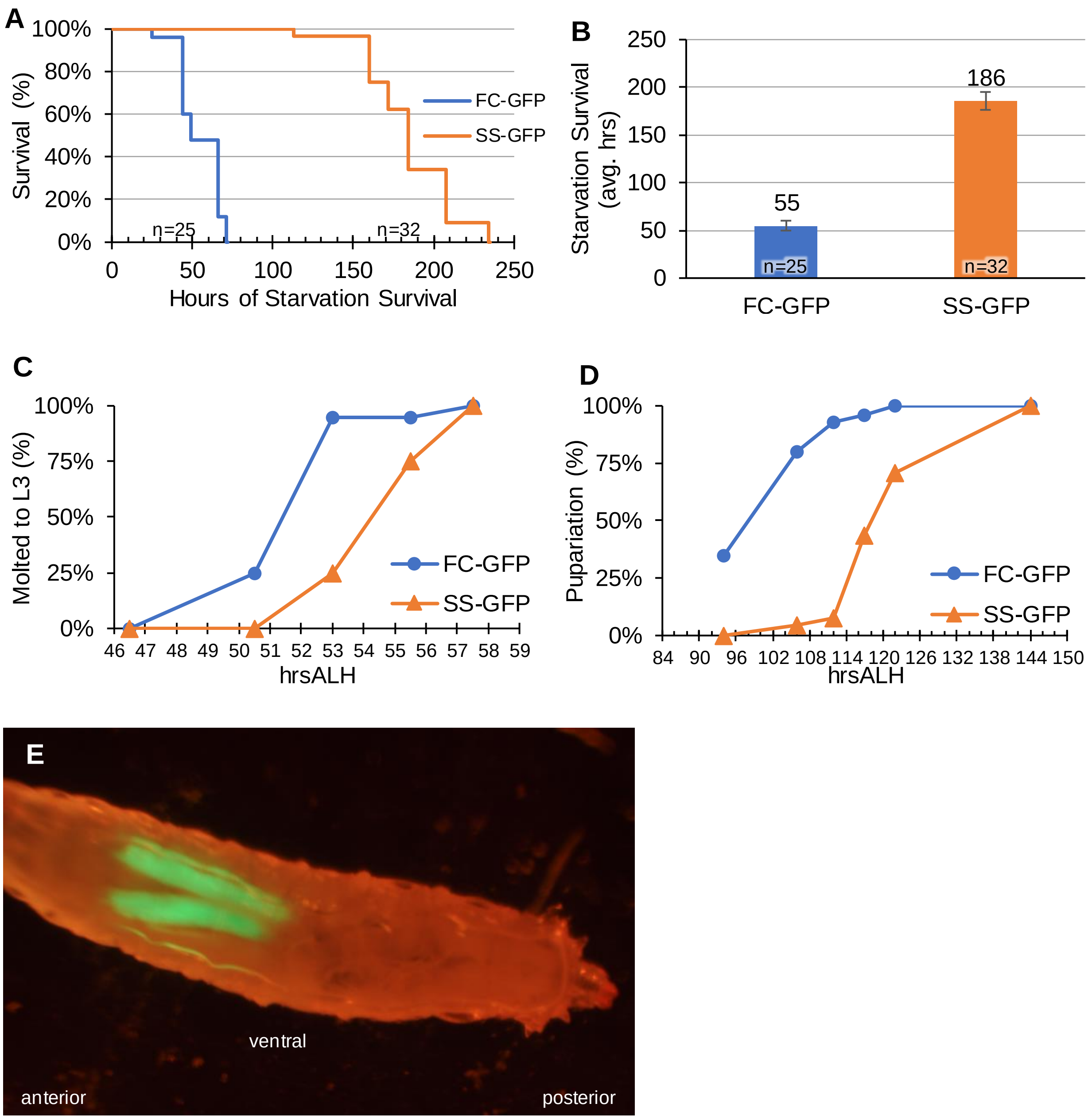

Fig. S2. Starvation-selected phenotypes are preserved in SS-GFP transgenic populations. (A) Kaplan-Meier survival curves for 4±1-day old transgenic populations. (B) Average starvation survival times represented by bars. Error bars represent 95% confidence interval of the mean. Scatter plots show representative experiments of (C) time to L3 molt and (D) time to pupariation where data points represent percent of animals having progressed to the next developmental stage, N=20 per population per time point. (E) Representative fluorescent dissecting microscope image of SS-GFP larvae expressing SgsΔGFP in the salivary glands.

Journal of Experimental Biology • Supplementary information

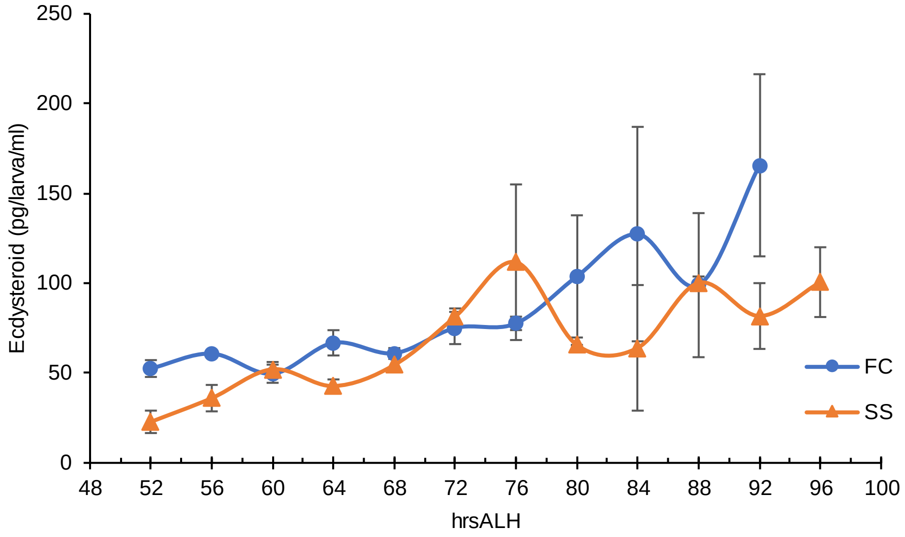

Fig. S3. Ecdysteroid Levels in Early Third Instar

Data points represent the average of three biological replicate values for the 20E EIA. Three groups of N=20 for time points 52-80 h ALH and N=10 for 84-96 h ALH were measured at each time point for each population. Results reported in pg per larva. Error bars represent standard error. All points including the apparent "peaks" when compared to the troughs are not of statistical significance (P>0.05).

Journal of Experimental Biology • Supplementary information

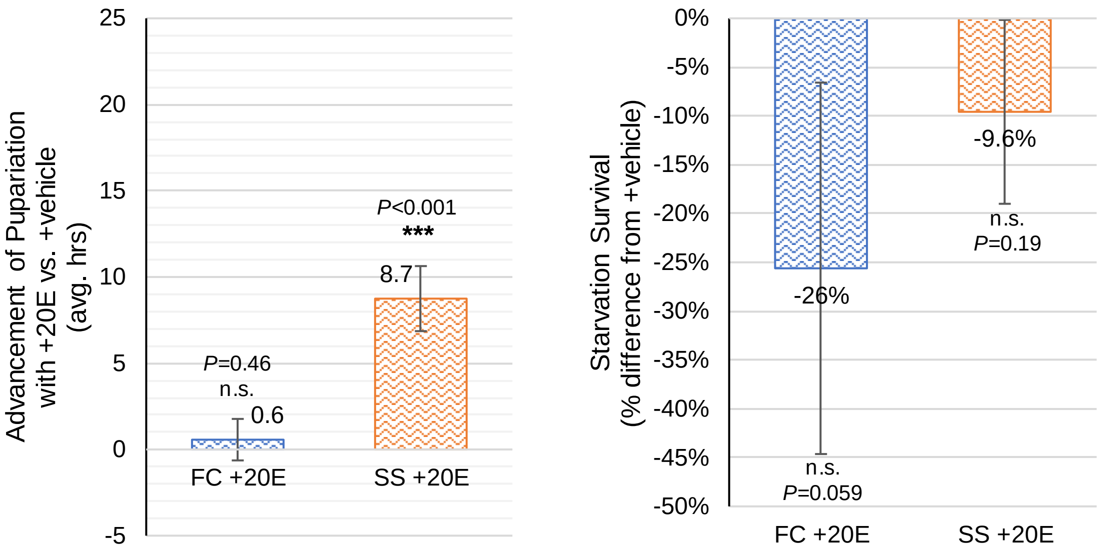

Fig. S4. Feeding Exogenous 20E

(A) Bar graph showing average hours of advancement of pupariation for animals fed on 20E supplemented food at 60hrsALH versus food with vehicle alone added from three independent experiments FC N=113 and 221 and SS N=122 and 241 for +20E or +vehicle alone, respectively.

Supplementary Materials and Methods

20E Feeding

Third instar SS and FC larvae synchronized at hatching were aged on normal food at 25°C under constant light in the manner described above. At 60hrsALH, in triplicate per population and treatment, 25 larvae were collected from the food and transferred to a vial with either 2mL of food containing 20E (0.54mg/mL) in 1ml 5% ethanol (vehicle) or vehicle alone, on top of 10mL of agar (to prevent the small volume of food from drying out). The treatment concentration of 20E used was empirically determined by Reynolds (2013) as the amount required to induce pupariation in the SS population (2x the conc. that rescues ecdysone-deficient mutants). Vials were assayed for the time to pupariation and then newly eclosed adults were collected and subjected to a starvation resistance assay or frozen for subsequent fat content or protein content assays.

Supplementary References

Reynolds, L. A. (2013). The Effects of Starvation Selection on Drosophila Melanogaster Life History and Development. *PhD Thesis*. University of Nevada, Las Vegas.

Journal of Experimental Biology • Supplementary information

Table S1. Cross-reactivity of various ecdysteroids with the Cayman Chemical 20E EIA Kit. Reproduced from Cayman Chemical, citing Porcheron et al., 1989.

Ecdysteroid	Reactivity with 20E EIA
**20-hydroxy-ecdysone**	100%
**Ecdysone**	100%
**2-deoxy-20-hyrdroxy-ecdysone**	88%
**Polypodine B**	70%
**2-deoxy-ecdysone**	63%
**Ponasterone A**	43%
**Cyasterone**	5%
**Podecdysone C**	4.5%
**Makisterone A**	4%
**26-hydroxy-ecdysone**	1.4%
**Muristerone A**	1.2%
**Kaladasterone**	1%
**22-epi-ecdysone**	<0.1%
**Posterone**	<0.1%

Journal of Experimental Biology • Supplementary information
